# Supplementary material for: Glecaprevir–pibrentasvir for chronic hepatitis C: Comparing treatment effect in patients with and without end-stage renal disease in a real-world setting
Source: PLoS One. 2020 Aug 13;15(8):e0237582. doi: 10.1371/journal.pone.0237582 (PMC7425913; doi:10.1371/journal.pone.0237582)
Supplement: S1 Data — (PDF) [file pone.0237582.s001.pdf]

Side-Effect-GOT	Side-Effect-GPT	Side-Effect-Bil(T)	Side-Effect-Anem	Anorexia	Dizziness
0	0	0	G0		
0	0	0	G0		
0	0	0	G0		
0	0	0	G1		
0	0	0	G2		
0	0	0	G0		
0	0	0	G0		
0	0	0	G1		
0	0	0	G1		
0	0	0	G0		
0	0	0	G1		
0	0	0	G1		
0	0	0	G0		
0	0	0	G1		
0	0	0	G0		
0	0	0	G0		
0	0	4.24	G0		
0	0	0	G0		
0	0	0	G2		
0	0	1.4	G0		
0	0	0	G0		
0	0	0	G0		
0	0	0	G2		
0	0	0	G0		
0	0	0	G0		
0	0	1.78	G0		
0	0	0	G0		
0	0	0	G2		
0	0	0	G0		
0	0	0	G1		
0	0	0	G0		
0	0	0	G0		
0	0	1.5	G0		
0	0	0	G1		
0	0	0	G0		
0	0	0	G0	1	
0	0	1.61	G0		
0	0	2	G2		
0	0	0	G2		
0	0	1.7	G3		
0	0	0	G2		
0	0	4.7	G2		
0	0	0	G0		
0	0	1.6	G0		
0	0	0	G0		
0	0	0	G1		
0	0	0	G1		
0	0	0	G0		
0	0	0	G0		

0	0	0	G1	
0	0	0	G0	
0	0	0	G0	
0	0	2	G0	
0	0	0	G0	
0	0	1.9	G2	
0	0	0	G0	
1.5	0	0	G0	
0	0	0	G2	
0	0	1.3	G0	
0	0	0	G0	
0	0	0	G1	
0	0	1.47	G0	
0	0	0	G2	
0	0	0	G0	
0	0	1.95	G0	
0	0	0	G0	
0	0	1.79	G1	
0	0	0	G0	
0	0	0	G0	
0	0	0	G0	
1.2	1.175	1.6	G0	1
0	0	0	G0	
0	0	0	G1	
0	0	0	G0	
0	0	0	G0	
0	0	0	G0	
0	0	0	G1	
0	0	0	G0	
0	0	0	G0	
0	0	0	G0	
0	0	0	G0	
0	1.25	1.46	G0	
0	0	0	G0	
0	0	1.79	G0	
0	0	1.33	G0	
0	0	0	G1	
0	0	0	G0	
0	0	0	G1	
0	0	0	G0	
0	0	1.2	G0	
1.125	1.25	0	G0	
0	0	0	G0	
0	0	1.7	G0	
0	0	0	G0	
0	0	2.23	G2	
0	0	0	G0	
0	0	2.93	G0	
0	0	0	G3	
1.95	1.7	0	G0	

0	0	0	G0		
0	0	0	G0		
0	0	0	G1		
0	0	0	G0		
0	0	1.6	G0		
0	0	0	G0		
0	0	0	G2		
0	0	1.67	G0		
0	0	2.76	G1		1
0	0	1.29	G0		
0	0	2	G0		1
0	0	0	G0		1
0	0	0	G1		
0	0	0	G2		
0	0	0	G1		
0	0	0	G0		
0	0	0	G0		
0	0	0	G0		
0	0	0	G0		
0	0	0	G0		
0	0	0	G0		
0	0	0	G0		
0	0	0	G0		
0	0	0	G0		
0	0	0	G0		
0	0	0	G0		
0	0	0	G0		
0	0	0	G0		
0	0	0	G2		
0	0	0	G1		
0	0	0	G2		
0	0	0	G0		
0	0	0	G0		
1.7	1.65	2.04	G0		
0	0	0	G0		
0	0	0	G0		
0	0	0	G1		
1.2	1.8	0	G2		
0	0	0	G0		
0	0	0	G1		
0	0	0	G0		
0	0	0	G0	1	1
0	0	0	G0		
0	0	0	G0		
0	0	0	G0		1
0	0	0	G0		
5.35	4.35	0	G0		
0	0	0	G1		
0	0	0	G0		
0	0	1.6	G3		

0	0	0	G0		
0	0	0	G0		1
0	0	0	G0		
0	0	0	G0		
0	0	0	G0		
0	0	0	G0		
0	0	1.9	G0		
0	0	0	G0		
0	0	0	G0		
0	0	0	G0		
0	0	0	G0		
0	0	1.8	G0		
0	0	0	G2		
0	0	0	G0		
0	0	0	G0		
0	0	0	G0		
0	0	0	G1		
0	0	0	G0		
0	0	0	G0		
0	0	0	G2		
0	0	0	G0		
1.675	1.85	0	G2	1	
1.55	0	1.4	G0		
0	0	1.9	G0		
0	0	0	G1		
0	0	0	G2		
0	0	0	G0		
0	0	0	G0		
0	0	0	G0		
0	0	0	G0		
0	0	0	G0		
0	0	0	G0		
0	0	0	G1		
0	0	0	G0		
0	0	2.2	G0		
0	0	0	G0		
0.925	1.775	0	G0		
0	0	0	G0		
0	0	1.38	G0		
0	0	2.65	G1		
3.575	6.4	0	G0		
0	0	0	G0		
0	0	0	G1		
0	0	2.3	G0		
0	0	3.4	G1		
0	0	0	G3		
0	0	0	G0		
0	0	0	G2		
0	0	0	G0		
3.65	4.475	2.11	G0		

0	0	0	G0	
0	0	0	G0	
0	0	1.48	G0	
0	0	0	G0	
0	0	0	G0	
2.95	7.475	0	G0	
1.85	1.85	2.71	G0	
0	0	0	G0	
0	0	1.38	G0	
0	0	0	G0	
1.05	1.125	0	G0	
0	0	0	G0	
0	0	0	G0	
1.275	1.15	0	G0	
0	0	0	G1	
0	0	0	G0	
0	0	0	G0	
0	0	0	G0	
0	0	1.37	G0	
0	0	0	G0	
0	0	0	G1	
0	0	0	G0	
0	0	0	G1	
0	0	0	G0	
1	1.425	0	G0	
0	0	0	G0	
0.975	1.375	0	G0	
0	0	0	G0	
0	0	0	G0	
0	0	0	G0	
0	0	2.67	G1	
0	0	0	G0	1
0	0	0	G0	
0	0	0	G0	
0	0	1.4	G0	
0	0	0	G0	

Malaise	GERD	Insomnia	sex	age	BMI	CR	Genotype	INR	BIL
			F	72	20.44	0.68	2	0.94	0.5
1			F	72	25.11	0.79	2	0.89	0.63
			F	56	26.78	1.04	2	0.85	0.69
	1		M	56	25.16	0.8	1a	0.96	0.47
			F	76	23.19	2.74	1b	0.88	0.28
			F	53	19.65	0.51	1b	0.82	0.6
1			F	66	23.21	0.6	1b	0.96	0.69
			F	78	17.65	1	1b	1.3	1.27
			M	55	21.48	6.27	2	1	0.83
			F	54	20.5	0.63	1b	0.91	0.75
			M	56	32.45	9.39	1b	0.79	0.51
			F	72	23.23	2.15	1b	0.85	0.64
			F	70	27.23	0.68	2	0.93	0.46
			F	72	20.89	0.8	2	0.99	0.6
			F	74	22.32	0.75	2	1.06	0.7
			F	58	22.4	0.64	1b	0.9	0.9
			F	78	23.96	0.6	2	1.14	1.59
			M	48	25.34	1.2	1a	0.98	1.03
1			F	80	22.6	1.77	1b	0.95	0.54
			M	43	19.15	0.8	2	0.96	0.77
			F	64	28.16	0.65	1b	0.93	0.64
			M	72	28.73	1.42	2	0.88	0.73
			F	69	26.58	6.09	2	0.94	0.34
		1	F	64	23.56	0.58	1b	0.94	1.23
			F	79	23.23	0.78	6	1	0.55
			M	67	20.31	0.29	1b	0.95	1.7
			M	69	23.09	1.03	1b	1.02	0.78
			F	83	27.19	8.38	2a	0.95	0.61
			M	60	26.61	1.34	1b	0.89	1.21
			F	78	20.7	8.6	1b	0.87	0.59
			F	53	20.93	0.68	2	0.95	0.94
			M	74	20.69	1.01	2	0.96	0.52
			M	59	26.03	1.1	1b	0.91	0.9
1			M	69	26.02	3.19	1b	0.91	0.89
			M	67	26.82	0.6	2	0.89	0.4
			F	61	22.55	0.86	2	0.89	0.73
			F	73	21.04	0.85	2	0.96	0.69
			F	46	20	0.4	2	0.97	1.6
			F	80	25.78	8.43	1a	0.95	0.44
			F	74	21.76	0.8	2	1.11	0.73
			F	81	22.67	4.37	1b	1.02	0.47
		1	M	61	24.22	7.49	1b	1.01	0.84
			M	62	25.56	1.15	2	0.93	0.7
			F	62	26.13	0.74	2	0.97	1.34
			F	62	28.72	0.95	1b	0.94	0.46
			F	55	21.9	0.58	2	0.91	0.34
1			F	77	19.98	1.09	2	0.77	0.31
			M	49	21.26	1.17	1b	0.79	0.5
			F	85	20.13333	5.4	6	10.6	0.4

			M	69	20.52	10.36	2	1	0.6
			F	58	23.15	0.58	1b	0.87	0.56
			F	63	17.72	0.59	1b	0.99	1.54
			M	50	17.71	0.82	3	0.93	0.9
			M	75	25.08	3.54	2	0.87	0.27
			F	84	17.59	2.47	2b	1	0.8
			F	51	18.59	0.63	1b	0.96	0.6
			M	50	24.02	0.67	6	0.91	0.36
			F	79	25.33	7.68	1b	0.83	0.57
			M	50	23.36	0.92	3	0.98	1.07
		1	M	60	24.41	13.02	1b	0.89	0.62
			M	85	22.03	7.2	1b	0.91	0.69
			M	82	25.4	1.26	1b	0.94	0.43
			F	60	33.12	12.13	1b	1.07	0.55
			M	49	21.77	0.73	3	0.94	0.42
			M	24	27.3	0.82	2	0.9	1.12
			M	43	23.03	0.95	2b	1.02	0.67
			F	81	21.91	6.44	1b	0.9	0.66
			F	48	20.4	0.63	1b	0.99	0.47
			F	66	25.72	0.63	1b	0.91	0.54
			M	46	23.85	0.83	2	0.86	0.9
		1	F	63	26.5	0.63	1b	1.02	0.71
			F	64	26.67	0.79	2	0.89	0.4
			F	63	27.73	1.24	1b	1.12	0.46
			M	36	26.4	0.82	3	0.82	1.09
			F	59	23.32	0.75	1b	1.23	1.03
			F	44	23.53	0.72	1b	0.87	0.41
			F	60	21.41	11.6	2	0.87	0.34
			M	85	23.9	2.02	1b	1	0.65
			F	56	21.94	0.57	2	0.96	0.7
1			F	44	17.63	0.58	1b	0.93	0.78
			F	57	21.6	0.65	2	0.85	0.61
		1	M	43	29.31	0.75	1b	1	1.2
			F	78	24.03	0.65	2	0.92	0.81
			F	56	25.97	0.38	1b	0.91	0.74
			M	60	22.76	0.99	1b	0.96	1.02
			M	60	20.52	7.5	2	0.89	0.8
			F	68	22.19	0.57	2	0.99	0.5
			M	65	30.86	7.05	2	0.9	0.61
1		1	M	54	19.71	1.02	1b+2	0.86	0.92
			F	74	19.31	0.63	6	0.89	0.92
			M	65	23.8	0.42	2	0.98	0.55
1			F	60	20	0.48	2	1.04	0.37
	1		F	73	23.81	0.73	2	0.9	1.04
			F	81	23.5	0.65	1b	0.95	0.6
			M	82	21.23	7.63	1b	0.9	0.46
			F	35	21.09	0.76	3	1	1.03
			F	63	26.02	0.48	2a	1.04	1.86
		1	F	67	23.37	9.56	1b	0.83	0.43
			M	47	26.22	0.77	3	1.15	0.94

		F	65	20.08	0.61	1or6	0.85	0.87
	1	M	52	24.26	11.91	1a	0.89	0.45
		M	60	22.45	7.49	1a	0.87	0.65
		F	68	22.22	0.73	4	0.98	0.36
	1	F	53	18.18	6.34	2	0.76	0.47
	1	F	62	19.65	0.63	1b	0.81	0.59
		M	86	18.12	8.75	1b	0.89	0.62
		F	67	20	1.24	1b	1.01	0.81
	1	F	72	26.01	6.83	1b	0.91	0.5
		F	76	22.81	0.58	2	1.87	0.66
		M	57	22.41	0.87	1b	0.99	1.11
	1	F	45	28.19	0.6	6	1.04	1.11
		M	43	23.89	1.06	3	0.89	0.79
		F	79	22.97	7.56	2	0.85	0.39
		M	54	26.98	0.99	2	0.99	1.77
		F	44	17.91	0.64	2	0.96	0.4
		M	68	22.77	0.9	2	1.03	0.7
1		M	57	22.66	0.93	1b	0.95	0.5
		M	43	20.42	1.01	3	1.03	1.21
		F	63	25.48	0.52	1b	0.95	0.88
		F	61	21.5	0.78	1b	0.89	0.76
		F	56	26.29	0.76	2	0.84	0.65
		M	43	24.38	0.92	1b	0.94	0.46
		F	72	28.93	1.74	2	0.94	0.66
		M	58	26.02	1.2	2	0.89	0.41
		F	58	25.74	1.06	2	1.02	0.6
		F	61	23.51	0.62	1a	1.06	0.65
		F	66	19.14	0.6	2	0.98	0.94
		M	85	21.92	3.47	1b	0.9	0.71
		M	53	24.61	0.1	2	0.9	0.72
		M	66	24.22	7.82	2	1	0.52
		M	35	19.53	0.87	2	1	0.43
		F	62	23.34	0.52	2	0.84	0.79
		M	39	22.04	1.06	3	0.9	1.16
		F	49	34.17	0.76	2	0.88	0.78
		M	84	21.42	1.17	2	0.85	0.45
		F	68	29.33	8.6	2	0.95	0.57
		M	38	30.49	8.9	3	0.97	0.81
		M	49	24.68	0.89	3	0.94	0.86
		M	90	19.72	10.1	1	0.97	0.46
		M	53	25.61	0.93	6	0.93	0.8
		M	53	26.35	0.79	1a	0.87	1.22
		F	42	21.3	0.93	3	0.9	0.19
		M	56	23.14	0.73	1	0.91	0.56
		M	59	25.91	0.83	1b	1.04	0.38
		F	43	19.31	0.45	2	0.96	0.53
		F	68	18.49	5.5	1b	0.89	0.91
		M	71	30.12	10.43	2	0.94	0.34
1		M	49	19.58	0.66	1b	1.04	0.52
		F	44	21.43	0.74	3	0.91	0.52

		M	63	24.35	0.78	2	0.82	0.7
1	1	F	51	25.37	0.48	1b	1	0.69
		M	59	27.4	1.27	2	1.05	1.25
		F	65	23.23	0.5	2	0.97	0.78
1		M	51	24.17	10.72	2	0.88	0.4
		F	52	28.62	0.7	2	1.07	1.44
		M	63	23.63	0.94	1b	1.02	2.01
		F	63	23.14	0.62	2	0.88	0.73
		F	68	20.4	0.61	1b	0.91	0.36
		M	48	24.34	1.09	6	0.98	1.01
		F	46	19.92	0.63	2	0.94	0.95
		M	62	24.85	0.64	1b	0.95	1.1
		M	53	24.1	2.9	1b	0.91	0.49
		M	39	25.45	0.9	6	0.89	0.5
	1	F	61	25.74	0.81	2	0.86	0.81
		F	52	22.85	0.65	1b	0.91	0.95
		M	71	19.53	12.52	1b	0.91	0.35
		M	60	25.43	0.83	1b	0.99	0.9
		F	60	22.65	7.38	2	0.85	0.35
		F	76	25.24	2.27	2	0.89	0.46
		M	60	25.54	7.13	2	0.9	0.79
		M	71	20.7	10.77	1b	0.96	0.44
		M	58	24.89	1.18	6	0.91	1.32
		M	68	23.07	0.92	2	0.9	0.85
		F	41	18.14	0.64	3	0.88	0.67
		F	64	30.86	1.46	1b	0.89	0.2
		M	44	32.11	1.1	3	0.92	0.67
		M	64	29.41	0.75	1b	0.97	1.06
		F	53	27.21	0.62	2	0.97	0.47
		F	72	27.7	0.8	2	0.89	0.53
		F	44	20.2	0.7	1b	0.85	0.32
		M	37	27.04	0.61	2	0.88	0.34
		M	59	28.73	10.72	2	1.07	1.38
		F	60	29.3	0.64	1b	0.9	0.64
		M	42	28.41	1.41	2	0.94	0.7
		M	65	26.41	7.51	2	0.92	0.79
		M	40	34.31	1.09	2	0.96	1.18
		M	44	27.48	0.97	3	0.98	0.69
		F	61	18.13	0.7	1b	1	0.89
	1	M	58	34.77	1.24	1a	0.95	0.73
	1	M	61	24.22	0.78	1b+2	0.89	0.59
		F	53	19.42	0.67	1b	0.96	0.68
	1	M	41	25.45	14.92	6	0.98	0.41
		M	50	23.03	1.05	2	0.82	0.53
		M	60	17.4	1.01	2	1.01	0.66
		F	54	23.07	5.96	2	0.95	0.44
		F	50	21.7	0.79	2	1.12	1.1
		F	47	22.7	0.57	1b	1.03	0.82
		F	55	25.15	0.67	2	0.97	0.7
		M	53	31.24	1.18	3	1.06	0.71

	F	60	23.28	6.5	1b	0.95	0.78
	M	40	20.58	0.73	1b	0.92	0.67
	M	75	21.3	1.14	1b	0.98	0.7
	M	43	26.35	0.86	6	0.92	0.39
1	F	60	25.88	0.81	1b	1.04	0.54
	M	54	28.14	1.06	1b	0.99	0.8
	M	66	23.63	5.97	2	0.95	0.47
	F	68	22.72	0.51	2	1.05	1.07
	F	63	20.7	0.71	2b	0.94	0.41
	F	71	28.51	0.63	2	1.09	0.42
	M	74	22.94	1.07	1b	0.89	0.38
1	F	72	21.93	0.92	1b	0.97	1.69
	F	65	20.2	0.67	2	0.97	1.29
	M	49	25.46	0.75	1a	0.94	0.56
	M	66	18.49	3.97	1b	0.89	0.26
	F	55	30.06	0.55	2	0.87	0.63
	F	50	19.45	0.43	1b	1.03	0.96
1	F	68	26.55	0.59	2	1.01	0.48
	M	45	24.8	0.88	6	0.94	0.31
	F	63	21.02	0.77	1b	0.95	0.29
	M	85	19.42	9.04	1b	0.97	0.7
	F	56	26.22	0.66	1b	0.92	0.88
	M	67	34.5	9.31	1b	1.33	0.93
	F	51	20.7	0.62	2	0.83	0.6
	M	43	22.24	0.83	1b	0.85	0.73
	F	63	22.4	0.44	2	0.87	0.39
	F	61	21.36	0.59	1b	0.91	0.99
	M	29	26.36	0.78	2	0.91	0.71
	F	76	21.61	0.9	1b	0.83	0.85
	M	48	23.08	0.77	1b	0.88	0.9
	M	58	22.58	6.23	3	1.03	1.47
	M	61	23.71	1.33	1b	0.89	0.37
	M	50	24.34	1	2	0.96	0.6
	M	53	30.49	1.02	2	0.93	0.68
	M	58	19.4	1.01	1b+3	0.96	1.27
	M	31	22.18	0.92	6	0.82	0.34

ALB	ASCITES	HEP	Hb	SVR	Skin Itching at W4	Skin itchin	Skin itching at W12
3.9	1		12.7	2	0	0	0
3.9	1		15.2	3	1	0	0
4.3	1		14.2	3	0	0	0
4.4	1		13.5	3	0	0	0
3.6			9.3		0	1	0
3.9	1		13.9	3	0	0	0
4.1	1		14.1		0	0	0
2.4	1		11.1		0	0	0
4	1		13.7	3	0	1	0
4.1	1		14.4	3	1	0	0
3.3	1		10.3	3	1	1	1
4	1	1	10.4		0	0	0
4.3	1	1	13.3	3	0	0	0
4.7	1		11.9	3	0	0	0
3.8	1		13.4	3	0	0	0
4.7	1		14.8		0	0	0
3.6	1	1	13.2	3	0	0	0
4.1	1		16	3	0	0	0
3.1	1	1	8.8	3	0	0	0
4.4	1		15.1	3	0	0	0
3.9	1	1	14.3	3	0	0	0
4.2	1	1	11.1	3	0	0	0
3.5			8.5	3	1	0	0
3.8	1		14.3	3	1	0	0
4	1	1	13.6	3	1	1	0
4.2	1	1	14	3	0	0	0
4	1	1	15.3	3	1	0	0
2.6	1	1	9.9	3	1	0	0
4.7	1		14.3	3	0	0	0
3.8	1		11.5	3	0	0	0
4.3	1	1	13.1	3	1	0	0
3.6	1	1	14.7	3	0	0	0
4.4	1		15.3		1	0	0
4	1		13.4		0	0	0
4.4	1		13.8		0	0	0
4.1	1		13.8	3	0	0	0
4	1		12.1	3	0	0	0
4.3	1		9		0	0	0
2.8	1	1	9.1	3	1	1	0
3.7	1		7.8	3	0	0	1
2.7	1		7.8	3	0	0	0
2.8	1		13.8		1	1	0
4.3	1		15	3	0	0	0
4.7	1		14.5	3	0	0	0
3.6	1		12.7	3	0	0	0
4	1	1	10.5	3	0	0	0
3.5	1		11.2		0	0	0
4.6	1	1	14.7		0	0	0
2.8	1	1	11.6		1	1	1

3.5	1		10.8	3	1	1	0
4.4	1		14.3	3	1	0	0
4.6	1		13.4	3	0	0	0
4.1	1	1	14.4	3	0	0	0
3.8	1	1	9.6	3	1	1	1
4.3	1	1	10.1		0	0	0
4.2	1	1	13.7		0	0	0
3.9			16.1	3	0	0	0
2.9	1		10.1		0	0	0
3.7	1	1	16.6		0	0	0
3.6	1		13.4	3	1	0	0
3.5	1		12.6	3	1	0	0
4	1	1	14.1	3	0	0	0
3.1	2	1	9.4	3	0	1	0
4	1	1	13.4	3	0	0	0
4.4	1		15.7	3	0	0	0
4.2	1	1	16.4	3	0	0	0
3.3	1	1	11.3		0	0	0
4.1	1	1	12.9	3	0	0	0
3.7	1	1	13.8	3	0	0	0
4.2	1	1	15.1	3	0	0	0
3.6	1		14.7	3	1	0	0
3.4	1	1	12.5		0	0	0
3.1	1		10.5	3	0	0	0
4.1	1	1	17.5	3	0	0	0
3.1	1		11.2		0	0	0
4.1	1		13.4	3	0	0	0
3.5	1		10.8	3	0	0	0
3.9	1		13.6		0	0	0
4.5	1		13.9	3	0	0	0
4.4	1	1	13.2	3	0	0	0
4.2	1		12.6		0	0	0
4.6	1	1	15.3	3	0	0	0
3.9	1		12.7	3	0	0	0
4.2	1		11.1	3	1	0	0
4.3	1		17.2	3	1	0	0
3.4	1		13.2	3	1	0	0
4.3	1	1	13.8		0	0	0
3.9	1		12.6	3	1	1	0
3.7	1		17.1	3	0	0	0
4	1		13.4	3	1	0	0
3.8	1	1	14.5	3	0	0	0
4	1		12	3	0	0	0
4.2	1		15		1	0	0
4.2	1	1	13.7		0	0	0
3.2	1	1	9.1	3	1	1	0
4.2	1	1	13	3	0	0	0
3.9	1	1	15.4	3	0	0	0
2.4	1	1	7	3	1	0	0
3.6	1		14.3	3	0	0	0

4.1	1		14.7	3	0	0	0
3.1	1	1	15.2	3	1	0	0
3.5	1	1	12.8	3	1	1	0
4.3	1	1	13.4	3	0	0	0
3.6	1		15	3	1	0	0
4.3	1		12.2	3	1	0	0
2.9	1		10.5	3	0	0	0
3.9	1		11.2	3	0	0	0
3.8	1	1	9.2		1	1	1
4.1	1		13.4	3	0	0	0
4.6	1		15.9	3	0	0	0
4.7	1		14.5	3	1	0	0
4.2	1	1	13.2		0	0	0
3.1	1	1	10.6	3	0	0	0
4.6	1		15.7	3	0	0	0
4.4	1	1	10.6	3	1	0	0
3.4	1		12.4	3	0	0	0
3.9	1		16.8	3	0	0	0
4.3	1	1	15.3	1	0	0	0
3.9	1		12.6	3	1	0	0
4.3	1		14.6	3	1	0	0
3.8	1		14	3	0	0	0
4.4	1		15.4	3	0	0	0
3.5	1		12.1	3	1	0	0
4.7	1		15.5	3	1	0	0
3.6	1	1	11		0	0	0
4	1	1	13.8	3	0	0	0
4.4	1		14.3	3	0	1	1
4.1	1	1	11	3	0	0	0
3.8	1	1	13.5	3	0	0	0
4.2	1		8.9	3	0	0	0
3.9	1		13.9	3	0	0	0
4.3	1	1	13.7	3	0	0	0
4.1	1		16.3	3	0	0	0
3.9	1		13.5		1	0	0
3.9	1		10.8	3	1	0	0
3.2	1		11.3	3	1	0	0
3.4	1		9.8	3	0	0	0
4.1	1		15.5	1	0	0	0
3.5	1	1	8.6	3	1	0	0
4.5	1	1	15	3	1	0	0
4.5	1	1	16.1	3	0	0	0
4.2	1	1	15	3	0	0	0
3.7	1		13.9	3	0	0	0
4	1		13.5	3	0	0	0
3.9	1		12.8	3	0	0	0
3.2	1	1	10.3	3	0	0	0
3.2	1		9.8	3	0	0	0
4.2	1		13.7	3	0	0	0
3.4	1		6.7	3	0	0	0

3.8	1		12.6	3	0	0	0
4.2	1	1	13.6	3	0	0	0
4.5	1		14.6	3	0	0	0
4.3	1	1	13.1		0	0	0
4.3	1		11.1	3	1	0	0
2.8	1		12.4	3	1	0	0
4.3	1	1	15.4	3	1	1	0
3.8	1	1	12.7		1	0	0
4.4	1		14.3	3	0	0	0
3.8	1	1	15.2		0	0	0
4	1	1	11.9	3	0	0	0
4.2	1		14.5		0	0	0
3.3	1		8.3	3	0	0	0
4.3	1		15		0	0	0
4.3	1		15.4	3	0	1	0
4.3	1		13.9	3	0	0	0
3.1	3		11	3	0	0	0
4.3	1	1	15.1		0	0	0
3.7	1		11.8	3	0	0	0
3.7	1	1	11.2		1	0	0
4	1		11.3	3	0	0	0
3.3	1	1	10.5	3	0	0	0
4.2	1		14.8	3	0	0	0
3.9	1		14.3	3	0	0	0
3.8	1	1	11.4	3	0	0	0
4.3	1		10		0	1	0
4.2	1	1	16.5	3	0	0	0
4.3	1	1	15.7	3	0	0	0
4.5	1		13.4	3	0	0	0
4.4	1		12.8		1	0	0
4	1		13		0	0	0
3.7	1		14.3	3	0	0	0
3.8	1		10	3	0	0	0
4.2	1	1	14.3	3	0	0	0
4.2	1		15.3		0	0	0
3.5	1		12.9	3	1	0	0
4.3	1	1	15	3	1	0	0
4.5	1		16.9	3	0	0	0
4.7	1	1	14.3	3	0	0	0
3	1	1	13.4		0	1	0
4	1		15.6		1	0	0
4.1	1		13.3	3	1	0	0
3.9	1		10.4	3	1	0	0
4.5	1		15.5	3	0	0	0
4.3	1		9.3		1	0	0
3.7	2		8.4	3	1	0	0
4.3	1		13.6	3	1	0	0
3.7	1		8.6	3	1	0	0
4.3	1	1	14.5		0	0	0
3.7	1	1	14.6	3	0	0	0

3.5	1	1	11.5		1	0	0
4.1	1	1	16.8	3	1	0	0
3.8	1		15.3	3	1	0	0
4.3	1	1	12.4	3	0	0	0
4.1	1		13.1	3	0	0	0
4.3	1		13.8		0	0	0
3.5	1		10.2	3	1	0	0
3.9	1		14	3	1	0	0
4.4	1	1	14.1	3	0	0	0
3.4	1		12.6	3	0	0	0
3.9	1		12.1	3	0	0	0
4	1		11.9	3	1	1	0
4.4	1	1	15.5	3	0	0	0
4.3	1	1	16.2	3	0	0	0
2	1		8.1	3	1	0	0
4.3	1		14.3	3	0	0	0
3.9	1		14.1	3	0	0	0
3.9	1	1	13	3	1	0	0
3.9	1		14.8	3	0	0	0
3.8	1		13.1	3	0	0	0
3.7	1	1	8.7	3	0	0	0
4.3	1		14.2	3	0	0	0
3.6	1		7.5	0	0	0	0
4.5	1		14.7		0	0	0
4	1		14.6		0	0	0
4.1	1		13.4	3	0	0	0
4	1	1	12.7	3	0	0	0
4.4	1		16.4	3	0	0	0
4.2	1	1	15	3	0	0	0
4.1	1	1	16.3	3	0	1	0
3.5	1	1	9.9	3	0	0	0
3.3	1		14		0	1	0
4	1	1	14.3		0	0	0
4.3	1		13.3		0	0	0
3.9	1		16.4		0	0	0
3.6	1		15.5		0	0	0

H/D	CAPD	H/D or CAPD
0	0	0
0	0	0
0	0	0
0	0	0
0	0	0
0	0	0
0	0	0
0	0	0
1	0	1
0	0	0
0	1	1
0	0	0
0	0	0
0	0	0
0	0	0
0	0	0
0	0	0
0	0	0
0	0	0
0	0	0
0	0	0
0	0	0
1	0	1
0	0	0
0	0	0
0	0	0
0	0	0
1	0	1
0	0	0
1	0	1
0	0	0
0	0	0
0	0	0
0	0	0
0	0	0
0	0	0
0	0	0
0	0	0
1	0	1
0	0	0
1	0	1
1	0	1
0	0	0
0	0	0
0	0	0
0	0	0
0	0	0
0	0	0
1	0	1

1	0	1
0	0	0
0	0	0
0	0	0
0	0	0
0	0	0
0	0	0
0	0	0
1	0	1
0	0	0
1	0	1
1	0	1
0	0	0
0	1	1
0	0	0
0	0	0
0	0	0
1	0	1
0	0	0
0	0	0
0	0	0
0	0	0
0	0	0
0	0	0
0	0	0
0	0	0
0	0	0
1	0	1
0	0	0
0	0	0
0	0	0
0	0	0
0	0	0
0	0	0
0	0	0
0	0	0
1	0	1
0	0	0
1	0	1
0	0	0
0	0	0
0	0	0
0	0	0
0	0	0
0	0	0
1	0	1
0	0	0
0	0	0
0	1	1
0	0	0

0	0	0
1	0	1
1	0	1
0	0	0
1	0	1
0	0	0
1	0	1
0	0	0
1	0	1
0	0	0
0	0	0
0	0	0
0	0	0
1	0	1
0	0	0
0	0	0
0	0	0
0	0	0
0	0	0
0	0	0
0	0	0
0	0	0
0	0	0
0	0	0
0	0	0
0	0	0
0	0	0
0	0	0
0	0	0
0	0	0
1	0	1
0	0	0
0	0	0
0	0	0
0	0	0
0	0	0
1	0	1
1	0	1
0	0	0
1	0	1
0	0	0
0	0	0
0	0	0
0	0	0
0	0	0
0	0	0
1	0	1
1	0	1
0	0	0
0	0	0

0	0	0
0	0	0
0	0	0
0	0	0
1	0	1
0	0	0
0	0	0
0	0	0
0	0	0
0	0	0
0	0	0
0	0	0
0	0	0
0	0	0
0	0	0
0	0	0
0	1	1
0	0	0
1	0	1
0	0	0
1	0	1
1	0	1
0	0	0
0	0	0
0	0	0
0	0	0
0	0	0
0	0	0
0	0	0
0	0	0
0	0	0
0	0	0
1	0	1
0	0	0
0	0	0
1	0	1
0	0	0
0	0	0
0	0	0
0	0	0
0	0	0
0	0	0
1	0	1
0	0	0
0	0	0
0	0	0
0	0	0
0	0	0
0	0	0
0	0	0

1	0	1
0	0	0
0	0	0
0	0	0
0	0	0
0	0	0
0	0	0
0	0	0
0	0	0
0	0	0
0	0	0
0	0	0
0	0	0
0	0	0
1	0	1
0	0	0
0	0	0
0	0	0
0	0	0
0	0	0
1	0	1
0	0	0
1	0	1
0	0	0
0	0	0
0	0	0
0	0	0
0	0	0
0	0	0
0	0	0
0	0	0
0	0	0
0	0	0
0	0	0
0	0	0
0	0	0
