# Supplementary material for: Glecaprevir–pibrentasvir for chronic hepatitis C: Comparing treatment effect in patients with and without end-stage renal disease in a real-world setting
Source: PLoS One. 2020 Aug 13;15(8):e0237582. doi: 10.1371/journal.pone.0237582 (PMC7425913; doi:10.1371/journal.pone.0237582)
Supplement: Attachment — Submitted filename: (Revision 1) Point to Point Respose.docx [file pone.0237582.s002.docx]

Dear Editor,

Thank you for reviewing our manuscript and providing your editorial comments as well as reviewers comments for improving our manuscript. Based on these comments, we have made several revisions to our manuscript, which we are hereby resubmitting for your consideration. Our point-by-point responses to the comments are detailed below.

Response to Editor’s comments

Editor:

1. Our internal editors have looked over your manuscript and determined that it is within the scope of our Liver Diseases Call for Papers. This collection of papers is headed by a team of Guest Editors for PLOS ONE. Additional information can be found on our announcement page: https://collections.plos.org/s/liver-diseases. If you would like your manuscript to be considered for this collection, please let us know in your cover letter and we will ensure that your paper is treated as if you were responding to this call. If you would prefer to remove your manuscript from collection consideration, please specify this in the cover letter.

Response: Thanks for your comment. We are glad the manuscript to be considered for Liver Diseases Call for Papers of PLOS ONE.

2. During our internal evaluation of the manuscript, we found minor instances of text overlap between your submission and the following previously published works:https://onlinelibrary.wiley.com/doi/abs/10.1111/jvh.13265 Please revise the manuscript to rephrase the duplicated text and cite your sources.

Response: Thanks for your comment. We revise the manuscript and cite the reference in the revised manuscript.

Response to Reviewer’s comments

*Reviewer: 1*

1. Please mention the reason why skin itching was higher in ESRD patients. Were they actually treatment-related or present before treatment?

Response: Thank for your comment. As mentioned in the discussion, previous clinical trials [2, 18] and real-world observations [1, 13, 21] have disclosed pruritus as the most frequent adverse event among patients with dialysis. Itching is constantly observed as the most common side effect for GLI/PIB based therapy. Our study is the first to describe the time course change of pruritus symptom. We found the majority patients experienced pruritus during the first four week of treatment (Table 4) and we believe it should be treatment related. We added Table 3 to describe other side effects of GLI/PIB therapy in our present study.

2. Were all patients treated at the same department in the hospital or were ESRD patients treated at a different department?

*Response*: Thank for your comment. In Taiwan, the use of DAA was restricted to licensed gastroenterologist and all the patients included treatment in the same department during the treatment period.

3. Please mention the reason for death in 3 patients.

Response: Thank for your comment. Unlike clinical trial, our report of real-world experience reflects the true patients. The cause of death was discussed in the discussion section in the revised manuscript. Two deaths occurred in the ESRD (n = 1) and non-ESRD (n = 1) groups, and both were super-elderly, 80 and 85 years old, respectively. Both patients died during sleep and had no signs of other systemic disease during the last phone contact. Cardiogenic cause of death was suspected and judged not to be related to the HCV treatment. The third patient died of chemotherapy related infection.

4. Please describe the method of HCV genotyping and HCV quantification.

Response: Thank for your comment. We used ART HCV assays (RealTime HCV and HCV Genotype II, Abbott Molecular, Abbott Park, IL, USA) to quantitatively measure HCV RNA concentrations and genotyping. We addressed this point in the revised manuscript.

5. Was there any possible reason why HCV RNA level in ESRD patients was lower than others?

Response: Thank for your comment. Previous reports (Kaiser T, et al. Kinetics of hepatitis C viral RNA and HCV-antigen during dialysis sessions: evidence for differential viral load reduction on dialysis. J Med Virol. 2008;80(7):1195-1201 ; Fabrizi F, et al. Kinetics of hepatitis C virus load during hemodialysis: novel perspectives. J Nephrol. 2003;16(4):467-475) have described a variable reduction of HCV-RNA during hemodialysis treatment sessions. Various mechanisms have been postulated such as adsorption of HCV onto dialysis membrane, HCV escape into spent dialysate, or destruction of HCV particles during dialysis.

6. Please include the treatment history in Table 1 and mention the treatment efficacy according to treatment history and treatment duration.

Response: Thank for your comment. We agree with your opinion that prior DAA treatment history is important to guide subsequent DAA therapy. As we mentioned efficacy and safety evaluation section, GLI/PIB was reimbursed by the National Health Insurance of Taiwan since Aug 2018 for patients with confirmed HCV viremia without prior use of DAA. Thus, there is no relevant treatment history for patients included in this analysis. We include the interferon therapy history in the revised Table 1. We will acknowledge it as our study limitation.

7. Please mention the presence of treatment history in relapsed patients. Was the drug resistance associated variants examined in these patients?

Response: Thank for your comment. We included only DAA treatment naïve patients in this study. Drug resistance is not tested prior to Glecaprevir–Pibrentasvir therapy in this study.

8. Line 52 “with oral DAAs”, there is error in font style.

Response: Thank for your comment. We correct this error in revised manuscript.

Reviewer #2: In this article, the author retrospectively evaluated treatment efficacy and safety of GLE/PIB in patients with HCV and end-stage renal disease. They showed similar SVR rate and higher prevalence of pruritus among patients with ESRD.

1) Treatment regimen should be described in more detail. The way how to decide treatment duration in Taiwan should be mentioned.

Response: Thank for your comment. GLI/PIB was reimbursed by the National Health Insurance of Taiwan since Aug 2018 for patients with confirmed HCV viremia without prior use of DAA. The treatment period was 8 weeks for patients without liver cirrhosis and 12 weeks for patients with compensated liver cirrhosis. The treatment period was 16 weeks for genotype 3 patients with prior interferon therapy

2) The number of patients who have experienced treatment with DAAs should be described.

Response: Thank for your comment. In Taiwan, the use of DAA was restricted to licensed gastroenterologist and all the patients included treatment in the same department during the treatment period. Patients with resistance to DAA therapy would be referred to special hepatology center and thus, we include only treatment naïve patients in this study. We will acknowledge it as our study limitation.

3) The relationship between death and treatment should be assessed.

Response: The cause of death was discussed in the discussion section in the revised manuscript. Two deaths occurred in the ESRD (n = 1) and non-ESRD (n = 1) groups, and both were super-elderly, 80 and 85 years old, respectively. Both patients died during sleep and had no signs of other systemic disease during the last phone contact. Cardiogenic cause of death was suspected and judged not to be related to the HCV treatment. The third patient died of chemotherapy related infection.

4) The complications other than pruritus should be described.

Response: Thank for your comment. We add Table 3 to mention other complications associated with GLI/PIB therapy in the revised manuscript.

Thank you for the opportunity to resubmit this manuscript for consideration for publication in the *PLOS One*. If you have any questions or comments regarding this manuscript, please do not hesitate to contact us using the details provided below.

Sincerely,

Hsu-Heng Yen M.D

Endoscopy Center

Division of Gastroenterology

Changhua Christian Hospital

Changhua City

Taiwan

Fax: +886-4-7228289

Tel: +886-4-7238595ext5501

E-mail: 91646@cch.org.tw
