# Supplementary material for: Glecaprevir–pibrentasvir for chronic hepatitis C: Comparing treatment effect in patients with and without end-stage renal disease in a real-world setting
Source: PLoS One. 2020 Aug 13;15(8):e0237582. doi: 10.1371/journal.pone.0237582 (PMC7425913; doi:10.1371/journal.pone.0237582)
Supplement: Attachment — Submitted filename: Rev 2 Point to Point .docx [file pone.0237582.s003.docx]

Dear Editor,

Thank you for reviewing our manuscript and providing your editorial comments as well as reviewers comments for improving our manuscript. Based on these comments, we have made several revisions to our manuscript, which we are hereby resubmitting for your consideration. Our point-by-point responses to the comments are detailed below.

Response to Reviewer’s comments

Reviewer #1: Because pruritus is common in dialysis patients, showing the prevalence of pruritus before treatment between groups will help to clearly understand the results of time course data in table 4.

Response: Thank for your comment. We believe this issue is important as the reviewer mentioned. The presence and the severity of pruritus at baseline is not a contra-indication for anti-HCV therapy in our real-world practice. As this is a retrospective real-world study, we didn’t record the pruritus symptoms before treatment initially in our practice. As mentioned in the discussion, after encountering the first patient with premature GLE/PIB therapy discontinuation from this side effect, we learned the importance of providing proactive measures to educate our patients before initiating therapy and using medications such as antihistamine and/or topical agent to relieve pruritus symptom therapy initiation. Such measures have helped patients adhere to the GLE/PIB therapy in previous studies. We acknowledge this as a limitation of the present study in the revised manuscript.

Thank you for the opportunity to resubmit this manuscript for consideration for publication in the *PLOS One*. If you have any questions or comments regarding this manuscript, please do not hesitate to contact us using the details provided below.

Sincerely,

Hsu-Heng Yen M.D

Endoscopy Center

Division of Gastroenterology

Changhua Christian Hospital

Changhua City

Taiwan

Fax: +886-4-7228289

Tel: +886-4-7238595ext5501

E-mail: 91646@cch.org.tw
