# Supplementary material for: Glecaprevir–pibrentasvir for chronic hepatitis C: Comparing treatment effect in patients with and without end-stage renal disease in a real-world setting
Source: PLoS One. 2020 Aug 13;15(8):e0237582. doi: 10.1371/journal.pone.0237582 (PMC7425913; doi:10.1371/journal.pone.0237582)
Supplement: Attachment — Submitted filename: Rev 3 Point to Point .docx [file pone.0237582.s004.docx]

Dear Editor,

Thank you for reviewing our manuscript and providing your editorial comments as well as reviewers comments for improving our manuscript. Based on these comments, we have made several revisions to our manuscript, which we are hereby resubmitting for your consideration. Our point-by-point responses to the comments are detailed below.

Response to ACADEMIC EDITOR’s comments

1) Authors should describe the definition of ESRD clearly.

Response: Thank for your comment. In the present study, we define ESRD as CKD Stage 5D who received dialysis (Kanda T, et al. Hepatol Int. 2019;13(2):103-9. )

Thank you for the opportunity to resubmit this manuscript for consideration for publication in the *PLOS One*. If you have any questions or comments regarding this manuscript, please do not hesitate to contact us using the details provided below.

Sincerely,

Hsu-Heng Yen M.D

Endoscopy Center

Division of Gastroenterology

Changhua Christian Hospital

Changhua City

Taiwan

Fax: +886-4-7228289

Tel: +886-4-7238595ext5501

E-mail: 91646@cch.org.tw
